# Microglia prevent beta-amyloid plaque formation in the early stage of an Alzheimer’s disease mouse model with suppression of glymphatic clearance

**DOI:** 10.1186/s13195-020-00688-1

**Published:** 2020-10-02

**Authors:** Weixi Feng, Yanli Zhang, Ze Wang, Hanrong Xu, Ting Wu, Charles Marshall, Junying Gao, Ming Xiao

**Affiliations:** 1 Jiangsu Province Key Laboratory of Neurodegeneration, Center for Global Health, Nanjing Medical University, Nanjing, China; 2 Brain Institute, The Affiliated Nanjing Brain Hospital of Nanjing Medical University, Nanjing, China; 3 Department of Neurology, The First Affiliated Hospital of Nanjing Medical University, Nanjing, Jiangsu China; 4 Department of Physical Therapy, University of Kentucky Center of Excellence in Rural Health, Hazard, KY USA

**Keywords:** Alzheimer’s disease, Aquaporin 4, β-Amyloid, Glia, Glymphatic system

## Abstract

**Background:**

Soluble beta-amyloid (Aβ) can be cleared from the brain through various mechanisms including enzymatic degradation, glial cell phagocytosis, transport across the blood-brain barrier, and glymphatic clearance. However, the relative contribution of each clearance system and their compensatory effects in delaying the pathological process of Alzheimer’s disease (AD) are currently unknown.

**Methods:**

Fluorescent trace, immunofluorescence, and Western blot analyses were performed to compare glymphatic clearance ability and Aβ accumulation among 3-month-old APP695/PS1-dE9 transgenic (APP/PS1) mice, wild-type mice, aquaporin 4 knock out (AQP4^−/−^) mice, and AQP4^−/−^/APP/PS1 mice. The consequence of selectively eliminating microglial cells, or downregulating apolipoprotein E (apoE) expression, on Aβ burden, was also investigated in the frontal cortex of AQP4^−/−^/APP/PS1 mice and APP/PS1 mice.

**Results:**

AQP4 deletion in APP/PS1 mice significantly exaggerated glymphatic clearance dysfunction, and intraneuronal accumulation of Aβ and apoE, although it did not lead to Aβ plaque deposition. Notably, microglia, but not astrocytes, increased activation and phagocytosis of Aβ in the cerebral cortex of AQP4^−/−^/APP/PS1 mice, compared with APP/PS1 mice. Selectively eliminating microglia in the frontal cortex via local injection of clodronate liposomes resulted in deposition of Aβ plaques in AQP4^−/−^/APP/PS1 mice, but not APP/PS1 mice. Moreover, knockdown of apoE reduced intraneuronal Aβ levels in both APP/PS1 mice and AQP4^−/−^/APP/PS1 mice, indicating an inhibitory effect of apoE on Aβ clearance.

**Conclusion:**

The above results suggest that the glymphatic system mediated Aβ and apoE clearance and microglia mediated Aβ degradation synergistically prevent Aβ plague formation in the early stages of the AD mouse model. Protecting one or both of them might be beneficial to delaying the onset of AD.

## Background

Alzheimer’s disease (AD) is one of the most common neurodegenerative diseases and is partially attributed to abnormal aggregation of oligomerized beta-amyloid (Aβ). Excessive accumulation of toxic forms of Aβ is mainly due to an imbalance between its production and clearance [1, 2]. Failure of Aβ clearance plays a crucial role in the etiology of AD, especially sporadic AD [3–5]. Therefore, exploring mechanisms and corresponding strategies of Aβ clearance will be a help in the prevention and treatment of AD.

A variety of clearance mechanisms, such as glial phagocytes, enzymatic degradation, and transport across the brain barriers, participate in the clearance of soluble brain Aβ peptides [6–8]. Recently, the glymphatic system, also known as the paravascular pathway, is implicated in clearance of a major portion of extracellular Aβ from the brain [9–11]. This route for Aβ clearance is mainly dependent on the bulk flow of interstitial fluid (ISF), mediated by astroglial aquaporin 4 (AQP4) [12–15]. AQP4, a water channel localized to perivascular end feet of astrocytes and ependymal cells, is responsible for rapid transport of water into and out of the brain parenchyma [16].

The glymphatic clearance malfunction is related to mislocalization of AQP4 due to reactive astroglosis in various mouse models, including brain aging [11], AD [17] and mild traumatic brain injury [18]. We have reported that knockout of the Aqp4 gene (AQP4^−/−^) aggravates brain Aβ accumulation and cognitive deficits of 12-month-old APP/PS1 mice [19]. AQP4-dependent glymphatic clearance potentially serves as a therapeutic target for abnormal aggregation of macromolecule-related neurodegenerative disorders including AD.

However, the consequences of glymphatic dysfunctions in the onset of AD and interactive roles of the abovementioned Aβ clearance systems in delaying or retarding AD progress are yet to be elucidated. In the present study, we compared glymphatic clearance ability among 3-month-old AQP4^−/−^/APP/PS1 mice, APP/PS1 mice, AQP4^−/−^ mice, and wild-type (WT) mice. We also investigated the consequence of selectively eliminating microglial cells or downregulating apolipoprotein E (apoE) expression on Aβ burden in the frontal cortex of AQP4^−/−^/APP/PS1 mice and APP/PS1 mice.

## Methods

### Animals

APP695/PS1-dE9 transgenic (APP/PS1) mice with a C57BL/6J background were obtained from Jackson Laboratories. These mice were bred with AQP4^−/−^ mice on a CD1 genetic background that were previously established in our laboratory [20]. F1 offspring were genotyped for APP and PS1 and for Aqp4 [19]. Female and male AQP4^+/−^/APP/PS1 mice were then mated to generate F2 offspring. AQP4^+/−^/APP/PS1 mice and AQP4^+/−^ mice were genotyped from these F2 littermates and interbred at 3 months of age to generate AQP4^+/+^ (WT) mice, APP/PS1 mice, AQP4^−/−^ mice and AQP4^−/−^/APP/PS1 mice. These mice were maintained until they reached 2 or 3 months of age for experiments. Animal experiments were performed in accordance with the recommendations in the Guide for the Care and Use of Laboratory Animals of Nanjing Medical University. All of the animals were handled according to approved by the Institutional Animal Care and Use Committee (NMU).

### Tracer injection

Anesthetized mice were fixed to a stereotaxic frame. For intracisternal tracer injection [13], the membrane of the cisterna magna was exposed and carefully pierced by a pulled glass capillary with a 10-μm tip diameter attached to a syringe. Then, 5 μl of 0.5% Texas Red-dextran (TR-d3, 3 kD, ThermoFisher, #D3328) dissolved in artificial cerebrospinal fluid (CSF) was injected, at a rate of 1 μl/min by a micropump (TJ-2A/L07-2A, Suzhou Wen Hao Chip Technology Co. Ltd., Jiangsu, China). For intrastriatal injection [13], the skin was opened and the skull was exposed. A burr hole was made with a hand-held drill on the coordinates: + 0.5 mm anterior/posterior, + 2.0 mm medial/lateral, and + 3.25 mm dorsal/ventral from bregma. The pulled glass capillary was implanted into the brain parenchyma. A 1 μl volume of 0.5% TR-d3 was then perfused, at a rate of 0.1 μl/min via the micropump. The micropipette remained in place for 5 min, then withdrew very slowly to avoid any possible backflow. Thirty minutes after intracisternal, or 1 h after intrastriatal injection, and with a second supplementary anesthesia, mice were transcardially perfused with fixative containing 4% paraformaldehyde (PFA) in 0.1 M phosphate buffer. The brains and deep cervical lymph nodes (dcLNs) were removed and post-fixed for 24 h.

### Cerebral injection of clodronate liposomes or adeno-associated virus (AAV) encoding apoE siRNAs

Clodronate liposomes (Liposoma, Amsterdam, The Netherlands; #P22JO419) were injected into the frontal cortex to deplete local microglia, as previously reported [21]. Three-month-old APP/PS1 mice and AQP4^−/−^/APP/PS1 mice were intracerebrally administrated 1.5 μl clodronate liposomes, or PBS liposomes, for 5 min (coordinates relative to Bregma: + 1.34 mm anterior/posterior, ± 1.00 mm medial/lateral, and + 1.00 mm dorsal/ventral). The microsyringe was withdrawn 2 min after injection. In order to downregulate expression of apoE in the frontal cortex, a single dose (10^10^ viral particles in 1.5 μl) of AAV encoding siRNAs targeting for apoE was administrated into the frontal cortex of 2-month-old APP/PS1 mice or AQP4^−/−^/APP/PS1 mice with the same stereotaxic parameters mentioned above. A subgroup of APP/PS1 mice and AQP4^−/−^/APP/PS1 mice received intracerebral injection of AAV encoding a non-sense sequence was used as controls. Mice were sacrificed for the Aβ-related pathological analyses, 5 days after clodronate liposome injection or 1 month after AVV injection, respectively.

### Section and tissue preparation

Following anesthesia, mice were transcardially perfused with 0.9% saline, followed by 4% paraformaldehyde (PFA) by perfusion pump (Cole-parmer, USA). Brain tissues and dcLNs were then post-fixed in 4% PFA overnight and dehydrated in 20% sucrose solution dissolved in 0.01 M PBS for 3 days. Sections were cut at 30 μm on a cryostat (Leica, Wetzlar, Germany) and mounted onto gelatin-coated slides. For CSF tracer experiments, PFA post-fixed forebrain tissues were coronally sliced on a vibratome (Leica) at 100 μm and mounted onto gelatin-coated slides in sequence. For biochemical analyses, anesthetized mice were euthanized by decapitation. The cerebral cortex was promptly dissected. Tissues were flash frozen in liquid nitrogen and stored at − 80 °C awaiting analysis.

### Immunofluorescence

Brain slices were blocked for 1 h at room temperature with 5% BSA, incubated with primary antibodies including rabbit anti-NeuN (1:500; Abcam, #ab177487), mouse anti-glial fibrillary acidic protein (GFAP) (1:800; Millipore, #AB3594), rabbit anti-GFAP (1:400; Abcam, #ab7260), rabbit anti-ionized calcium-binding adaptor molecule 1 (Iba1) (1:1000; Wako, #019-19741), rat anti-CD68 (1:100; bio-rad, #MCA1957), goat anti-lysosome-associated membrane glycoprotein 1 (Lamp1) (1:200, R&D systems, #AF4320), mouse anti-glutamine synthetase (GS) (1:100, BD, #610517), rabbit anti-Aβ_1–40_ (1:250; CST, #12990), mouse anti-total Aβ (Aβ_1–16_) (1:1000; Covance, # 803001), rabbit anti-AQP4 (1:400; Millipore, #AB3594), rabbit anti-apoE (1:200; Abcam, #ab20874), or mouse anti-apoE (1:200; Abcam, #ab1906) overnight at 4 °C. After extensive rinsing, sections were incubated for 2 h at room temperature with AF488-conjugated donkey anti-mouse IgG (1:1000, ThermoFisher, #A21202), AF488-conjugated donkey anti-rabbit IgG (1:1000, ThermoFisher, #A21206), AF555-conjugated donkey anti-rabbit IgG (1:1000, ThermoFisher, #A31572), AF555-conjugated donkey anti-mouse IgG (1:1000, ThermoFisher, #A31570), AF647-conjugated donkey anti-mouse IgG (1:1000, ThermoFisher, #A31571), or AF488-conjugated donkey anti-goat IgG (1:1000, ThermoFisher, #A11055). The sections were washed for 3 × 5 min in PBS containing 1.5 μM 4′,6-diamidino-2-phenylindole (DAPI, Invitrogen, #D21490), then cover slipped with buffered PBS/glycerol.

### Western blot

Cerebral cortex extracts were loaded onto 10–16% Tris/tricine SDS gels and transferred to nitrocellulose membranes before overnight incubation with one of the following primary antibodies: mouse anti-β-site amyloid precursor protein-cleaving enzyme 1 (BACE1) (1:1000; Millipore, #MAB5308), anti-PS1 (1:1000; Sigma-Aldrich, #PRS4203), anti-neprilysin (NEP) (1:1000; Millipore, #AB4348), anti-insulin-degrading enzyme (IDE) (1:1000; Abcam, #ab32216), anti-low-density lipoprotein receptor-related protein (LRP1) (1:1000; Abcam, #ab92544), anti-APP (C-terminal) (1:1000; Sigma-Aldrich, #A8717), mouse anti-total Aβ (Aβ_1–16_) (1:1000; Covance, #803001), and anti-apoE (1:1000; Abcam, #ab20874) or GAPDH (1:1000; Bioworld; #AP0063). Horseradish peroxidase-conjugated secondary antibodies (Vector Laboratories) were used, and bands were visualized using ECL plus detection system. GAPDH was utilized as an internal control for protein loading and transfer efficiency.

### Enzyme activity assay

The enzyme activities of NEP and IDE were detected by NEP and IDE activity assay kit, respectively (ANASPEC, #72223 and #72231). Briefly, the frontal cortex of mice was homogenized and the supernatants were collected for the following experiment. Purified NEP or IDE enzyme was used as positive control, and the fluorescence intensity was measured at Ex/Em = 490 nm/520 nm.

### Quantitative analyses of images

All fluorescence micrographs were captured by a digital microscope (DM4000B, Leica) with a constant exposure time, offset, and gain for each immunofluorescent staining marker and analyzed by the Image-Pro Plus 6.0 Analysis System (Media Cybernetics Inc., San Francisco, CA, USA). The frontal cerebral cortex in each section was manually delineated. The area of positive signal of GFAP, GS, Ibal, Aβ_1–40_, total-Aβ, thioflavine-S, or TR-d3 was measured using the interest grayscale threshold analysis with constant settings for minimum and maximum intensities for each staining marker [19]. The number of total-Aβ or thioflavine-S-positive plaques in the frontal cortex per section was quantified. The percentage area of positive signal was calculated by dividing the area of positive signal to the total area in the region of interest. The percentage area of TR-d3 in the dcLNs was also detected with the same method. The fluorescence intensity of TR-d3 in the cortex was determined along a corresponding linear region adjacent to the vessels as previously described [22]. For analysis of phagocytosis and lysosomal degradation of microglia, the percentage of area covered by double positive signal for CD68, or Lamp1, and Iba1 was calculated [14]. For analysis of total-Aβ (or apoE) accumulation within neurons, astrocytes, and microglia, the percentage of area covered by double-positive signal for total-Aβ (or apoE) and NeuN, GFAP, or Iba1 was calculated, respectively. The percentage of area covered by triple-positive signal for Lamp1, Aβ, and Iba1 was also analyzed in the images of the frontal cortex of AQP4^−/−^/APP/PS1 mice and APP/PS1 mice. For analysis of AQP4 polarization, the mean AQP4-immunoreactive intensity at the regions immediately abutting vessels or pia maters and correspondingly adjacent parenchymal domains was measured. AQP4 polarization was obtained by comparing expression ratios of AQP4 at perivascular or pia surface versus parenchymal domains [9, 19].

### Statistical analysis

All data were expressed as means ± SEM using SPSS software, version 16.0 (SPSS Inc., USA), and analyzed by Student’s *t* test or ANOVA followed by Newman-Keuls post hoc multiple comparison test as indicated in the figure legends.

## Results

### AQP4 deletion exacerbated impairment of glymphatic transport in 3-month-old APP/PS1 mice

**Fig. 1:**
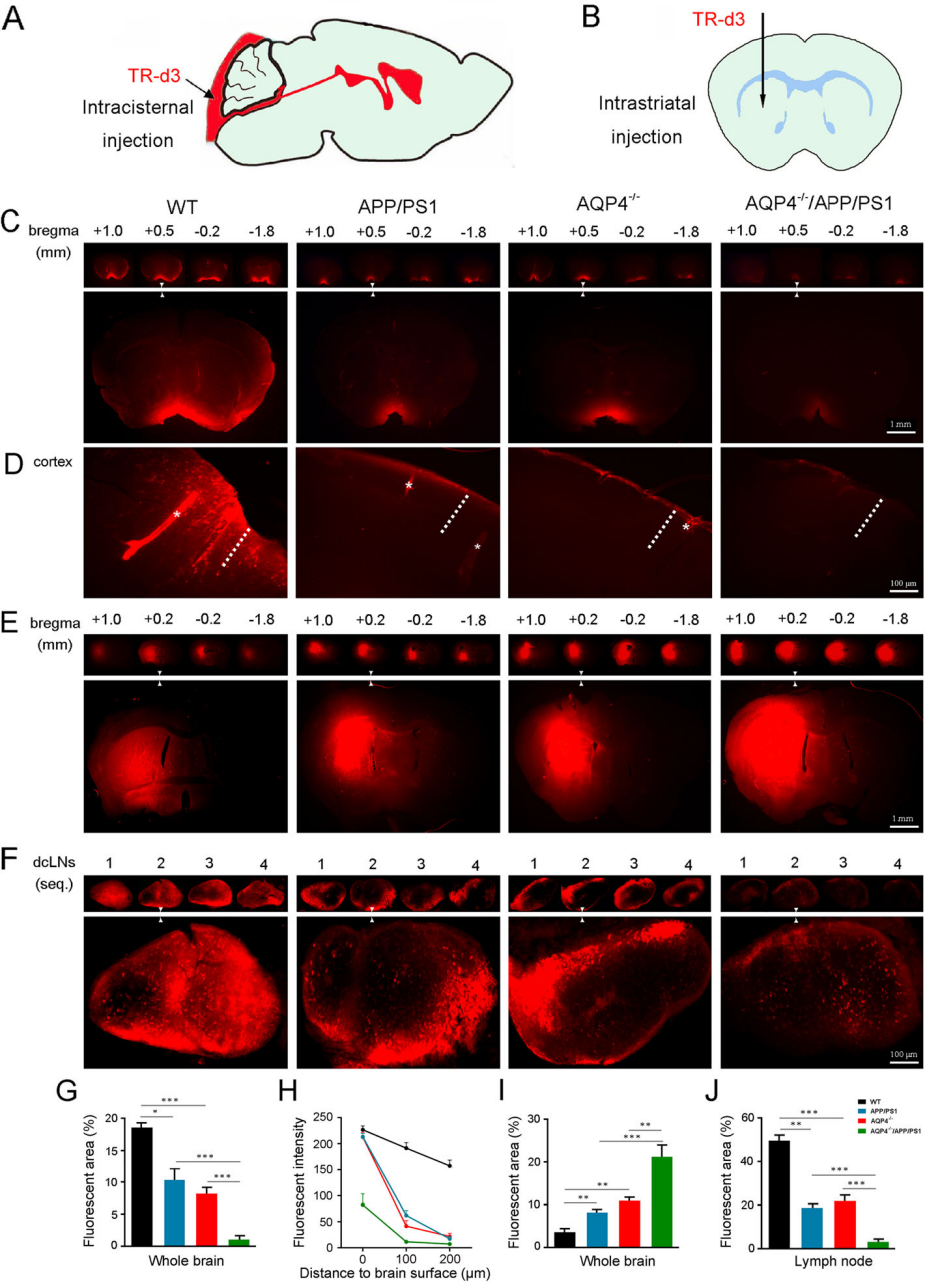
AQP4 deletion aggravates decreases of florescent tracer influx into, and efflux out of, the brain of 3-month-old APP/PS1 mice. **a**, **b** Schematic images for intracisternal injection and intrastriatal injection of TR-d3. **c** Representative images of serial coronal brain sections from 1.0 mm anterior to 1.8 mm posterior to bregma (upper panel) and high magnification micrographs of the sections at the level of 0.5 mm anterior to bregma (low panel) showing TR-d3 penetration into the brain parenchyma at 30 min after intracisternal injection. **d** High magnification micrographs of the cortex showing TR-d3 penetration into the perivascular space (star) and adjacent brain parenchyma (dotted line) at 30 min after intracisternal injection. **e** Representative images of serial coronal brain sections from 1.0 mm anterior to 1.8 mm posterior to bregma (upper panel) and high magnification micrographs of the sections at the level of 0.2 mm anterior to bregma (low panel) showing residue of TR-d3 within the striatum at 1 h after intracisternal injection. **f** Low (upper panel) and high (low panel) magnification micrographs of sections of the dcLNs showing TR-d3 distribution at 1 h after intracisternal injection. **g** Quantification of intracisternally injected TR-d3 area fraction in the brain. **h** Quantification of the mean fluorescence intensity of TR-d3 in the cerebral cortex after intracisternal injection. **i** Quantification of intrastriatally injected TR-d3 area in the brain. **j** Quantification of intrastriatal injected TR-d3 in the dcLNs. Data were analyzed by the two-way ANOVA with Tukey’s post hoc test or repeated two-way ANOVA with Tukey’s post hoc test. Data are mean ± SEM. *n* = 4 per group, *p < 0.05; **p < 0.01; ***p < 0.001

Previous studies reported that glymphatic transport is suppressed in both APP/PS1 mice [17] and AQP4^−/−^mice [9, 13, 15]. In our preliminary experiments, we demonstrated that AQP4 deletion in APP/PS1 mice does not induce spatial cognitive dysfunction at 3 months old (Additional file 1: Supplemental Methods, Additional file 2: Fig. S1a-f). Therefore, this age was chosen to investigate the consequence of AQP4 deletion in APP/PS1 mice on glymphatic function by examining fluorescent tracer TR-d3 (3 kDa) penetration into and clearance out of the brain (Fig. 1a, b). Thirty minutes after injection into the cisterna magna, parenchymal distribution of TR-d3 was less in both AQP4^−/−^ mice and APP/PS1 mice than WT mice (*p* < 0.001; *p* < 0.05, respectively). Notably, CSF tracer influx was further decreased in AQP4^−/−^/APP/PS1 mice compared to that of AQP4^−/−^ mice (*p* < 0.001) or APP/PS1 mice (*p* < 0.001) (Fig. 1c, g). As shown by high magnification micrographs of the frontal cortex (Fig. 1d), there was strong fluorescent intensity within the perivascular space and adjacent brain parenchyma in WT mice. However, there were only weak fluorescent signals in cortical parenchyma of AQP4^−/−^ mice and APP/PS1 mice. As for AQP4^−/−^/APP/PS1 mice, the fluorescent tracer was only observed at the outermost surface of brain parenchyma. Quantification of the TR-d3 fluorescent intensity as a function of the distance from the brain surface to the parenchyma further demonstrated that AQP4 deletion reduced glymphatic CSF influx in both WT mice and APP/PS1 mice (both *p* < 0.001) (Fig. 1h). Consistently, AQP4 deletion also suppressed macromolecule clearance from the brain to the peripheral lymphatic system. One hour after intrastriatal injection of TR-d3, AQP4^−/−^/APP/PS1 mice displayed a high percentage of fluorescent areas within the striatum (Fig. 1e, i), and a low percentage of fluorescent areas within the dcLNs, compared to AQP4^−/−^ mice (*p* < 0.01; *p* < 0.001, respectively) or APP/PS1 mice (both *p* < 0.001) (Fig. 1f, j).

### AQP4 deficiency increased accumulation of intraneuronal Aβ accumulation without causing extracellular plaque deposition in 3-month-old APP/PS1 mice

**Fig. 2:**
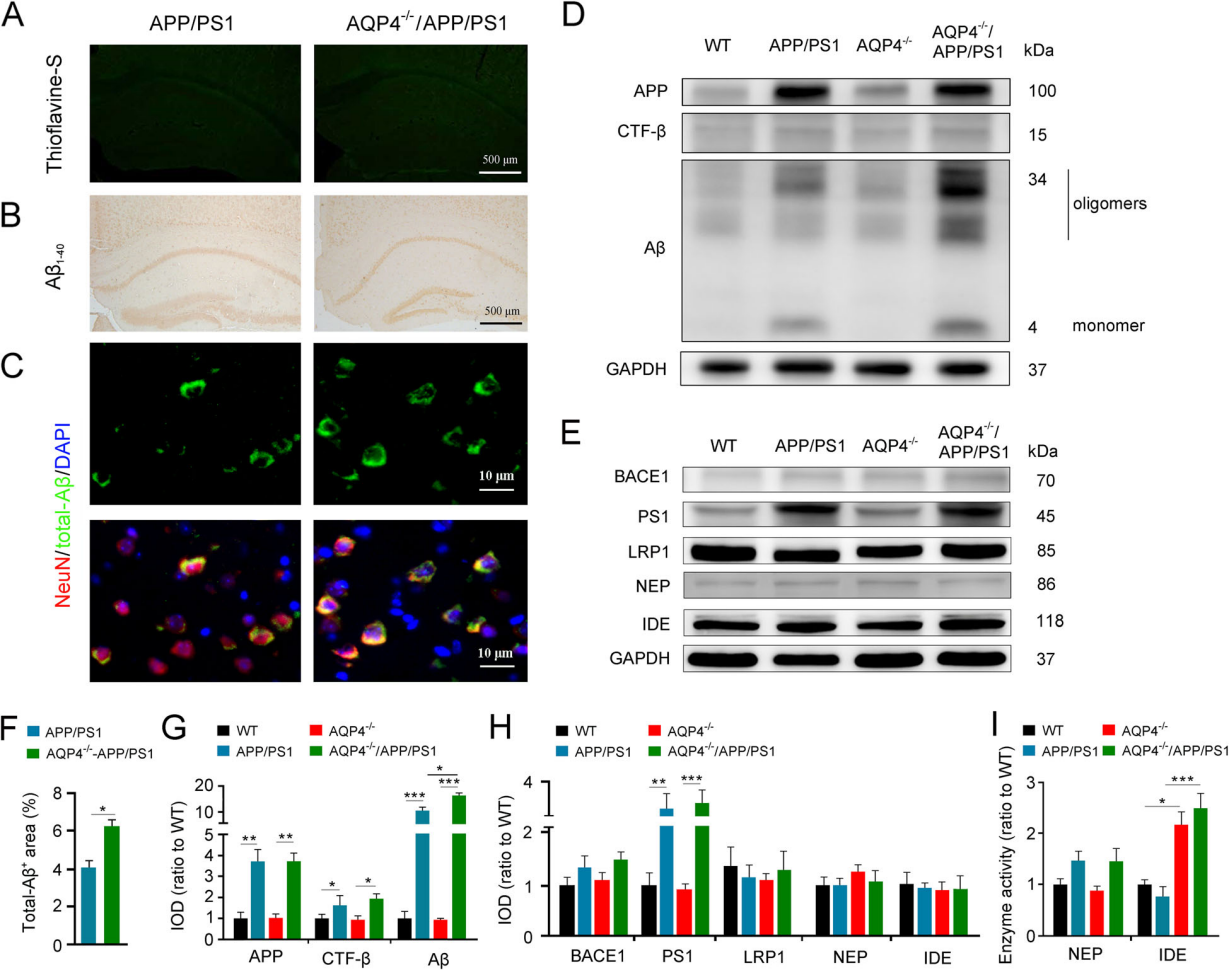
AQP4 deletion increased intraneuronal Aβ and IDE activity without causing plaque formation in the cerebral cortex of 3-month-old APP/PS1 mice. **a**, **b** Brain sections stained by thioflavine-S florescence and Aβ_1–40_ immunohistochemistry showing no Aβ plaque disposition. **c** Double immunofluorescence for total-Aβ and NeuN. There was increased Aβ immunostaining in cerebral neurons of AQP4^−/−^APP/PS1 mice compared to those in APP/PS1 controls. **d**, **g** Representative Western blot bands and densitometry analysis of APP, CTF-β, and Aβ monomer/oligomers. **e**, **h** Representative Western blot bands and densitometry analysis of BACE1, PS1, LRP1, IDE, and NEP in the cortex. **f** Quantification of Aβ-positive area fraction. **i** Enzyme activity assay of NEP and IDE. Data in **f** were analyzed by Student’s *t* test; other data were analyzed by the two-way ANOVA with Tukey’s post hoc test. Data are mean ± SEM. *n* = 4 per group. *p < 0.05; **p < 0.01; ***p < 0.001

We evaluated whether AQP4 deletion exacerbates impairments of glymphatic clearance, subsequently accelerating Aβ-related neuropathology of 3-month-old APP/PS1 mice. Neither thioflavine-S-positive plaques, nor Aβ_1–40_ positive plaques, were visible in the cerebral cortex of APP/PS1 mice and AQP4^−/−^/APP/PS1 mice (Fig. 2a, b). However, AQP4 deficiency in APP/PS1 mice intensified intraneuronal Aβ aggregation, as revealed by double immunofluorescent staining of NeuN and total-Aβ (Fig. 2c, f). Western blot results showed that the expression levels of Aβ monomer and oligomers were significantly increased in AQP4^−/−^/APP/PS1 mice (*p* < 0.05), but APP and its intracellular β-C-terminal fragment (CTF-β) were not changed when compared with those in APP/PS1 mice (Fig. 2d, g). AQP4 deficiency did not affect expression of β-secretase, identified as BACE1, and γ-secretase, a putative enzymatic complex containing PS1, in the cortex of WT mice and APP/PS1 mice. Expression levels of LRP1, a functional protein for transport of brain Aβ across the brain-blood-barrier, were also comparable among the four genotype groups. Transgenic APP/PS1 and/or AQP4 deletion did not affect protein levels of NEP or IDE, two main enzymes responsible for Aβ degradation. (Fig. 2e, h). However, the enzyme activity of IDE, but not NEP, was upregulated in AQP4^−/−^/APP/PS1 mice, when compared that in APP/PS1 mice (*p* < 0.001) (Fig. 2i).

### Eliminating reactive microgliosis increased Aβ aggregation in the cerebral cortex of 3-month-old AQP4^−/−^/APP/PS1 mice

**Fig. 3:**
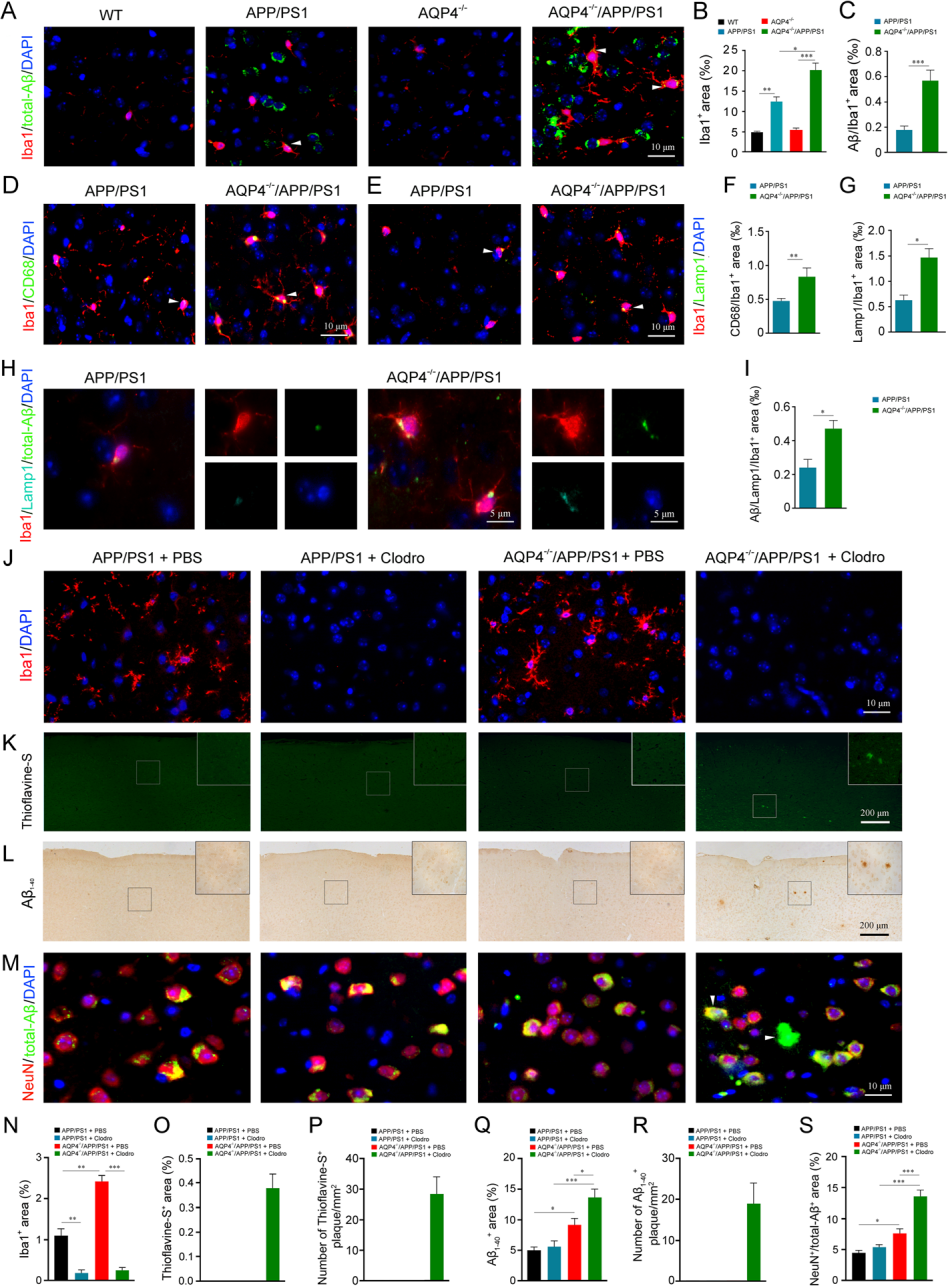
Increased microglial activation, and Aβ plaque deposition following eliminating microglia in AQP4^−/−^/APP/PS1 mice. **a** Double immunofluorescence for total-Aβ and Iba1. Microglia were apparently activated in the cerebral cortex of AQP4^−/−^/APP/PS1 mice, compared to those in APP/PS1 controls. Dot-like signals of total-Aβ (arrowheads) were frequently observed in AQP4^−/−^/APP/PS1 microglial cells. **b**, **c** Quantification of Iba1-positive, and iba1 and total-Aβ double-positive area fraction in the cerebral cortex, respectively. **d**, **f** Double immunofluorescence and quantification for CD68 and Iba1. CD68-positive microglia (arrowheads) were apparently increased in the cerebral cortex of AQP4^−/−^/APP/PS1 mice, compared to those in APP/PS1 controls. **e**, **g** Double immunofluorescence and quantification for Lamp1 and Iba1. Lamp1-positive microglia (arrowheads) were also apparently increased in the cerebral cortex of AQP4^−/−^/APP/PS1 mice. **h**, **i** Triple immunofluorescence and quantification for Lamp1, total-Aβ, and Iba1. Aβ immunoreactive products were restrictively localized to Lamp1-positive lysosome of Iba1-positive microglia. **j** Iba1 positive microglia were almost eliminated in both APP/PS1 mice and AQP4^−/−^/APP/PS1 with clodronate liposome treatment. **k**, **l** Brain sections stained by thioflavine-S and Aβ_1–40_, respectively. Aβ plaque disposition was present at the cerebral cortex of AQP4^−/−^/APP/PS1 mice received local injection of clodronate liposomes. **m** Double immunofluorescence for total-Aβ and NeuN. Total-Aβ immunoreactivity (arrowheads) was increased within the cytoplasm of NeuN positive neurons of AQP4^−/−^/APP/PS1 mice injected clodronate liposomes. **n**–**s** Quantification of Iba1, thioflavine-S, Aβ_1–40_, and total-Aβ-positive area fraction or number in the cerebral cortex, respectively. Data in **c**, **f**, **g**, and **i** were analyzed by Student’s *t* test and in **b**, **n**, **q**, and **s** were analyzed by the two-way ANOVA with Newman-Keuls post hoc test. Data are mean ± SEM, *n* = 4 per group, *p < 0.05; **p < 0.01; ***p < 0.001

Secreted IDE in the brain is mainly produced by microglia [23]. Increased IDE activity might be involved in no extracellular Aβ plaque formation in 3-month-old AQP4^−/−^/APP/PS1 mice. To test this possibility, we analyzed the effect of AQP4 deletion on the activation of microglia in the early pathological stage of APP/PS1 mice. AQP4^−/−^/APP/PS1 mice showed more obvious activation of microglia in the frontal cortex, as revealed by a high percentage area of Iba1 immunostaining (*p* < 0.05, versus APP/PS1 mice; Fig. 3a, b). However, both AQP4^−/−^/APP/PS1 mice and APP/PS1 mice had mild activation of astrocytes with a similar percentage area of GFAP or GS immunopositive signals in their frontal cortex (Fig. S2a-d). Consistent with astrocyte activation, AQP4 expression was slightly but widely increased in the brain parenchyma of APP/PS1 mice, impairing its specific localization abutting microvessels and pia maters (Fig. S3a, b).

Microglia and astrocytes participate in uptake and phagocytosis of ISF Aβ [24, 25]. We investigated the effect of AQP4 deletion on these processes in APP/PS1 mice. AQP4^−/−^/APP/PS1 mice exhibited more Aβ aggregation within the cytoplasm of Iba1-positive microglia than APP/PS1 mice (*p* < 0.001; Fig. 3a, c). However, in GFAP-positive astrocytes, Aβ immunoreactive products were negligible in both AQP4^−/−^/APP/PS1 mice and APP/PS1 mice (Fig. S2a, b). These results suggest that microglia, rather than astrocytes, increase uptake of ISF Aβ during the early pathological stages of AQP4^−/−^/APP/PS1 mice.

Microglial phagocytosis of Aβ may offset impairment of glymphatic clearance of Aβ caused by AQP4 deletion. To test this possibility, microglia expressing CD68 and Lamp1, markers of phagocytosis and lysosomal degradation [22] were examined. AQP4^−/−^/APP/PS1 mice showed a higher percentage of area covered by CD68/Iba1 or Lamp1/Iba1 double-positive signals in the frontal cortex than APP/PS1 mice (*p* < 0.01, *p* < 0.05, respectively; Fig. 3d–g). Moreover, Lamp1/Aβ/Iba1 triple immunostaining revealed that Aβ in the lysosome of microglia was also increased in AQP4^−/−^/APP/PS1 mice (*p* < 0.05; Fig. 3h, i), indicating increased phagocytic ability of Aβ by activated microglia.

To verify this speculation, clodronate liposomes, which specifically depletes macrophages [20, 26, 27], were injected into the frontal cortex to locally eliminate microglia (Fig. 3j, n). Both thioflavine-S-positive plaques and Aβ_1–40_-positive plaques were clearly observed in the frontal cortex of AQP4^−/−^/APP/PS1 mice, but not APP/PS1 mice, 5 days after treatment of clodronate liposomes (Fig. 3k, l, o–r). AQP4^−/−^/APP/PS1 mice injected with clodronate liposomes also noticeably improved accumulation of intracellular Aβ (*p* < 0.001, versus PBS controls; Fig. 3m, s). In addition, astrocytes in the frontal cortex underwent elevated activation in both APP/PS1 mice and AQP4^−/−^/APP/PS1 mice that received injection of clodronate liposomes (Fig. S4a, b).

### Increased apoE levels in the brain of 3-month-old AQP4^−/−^/APP/PS1 mice

**Fig. 4:**
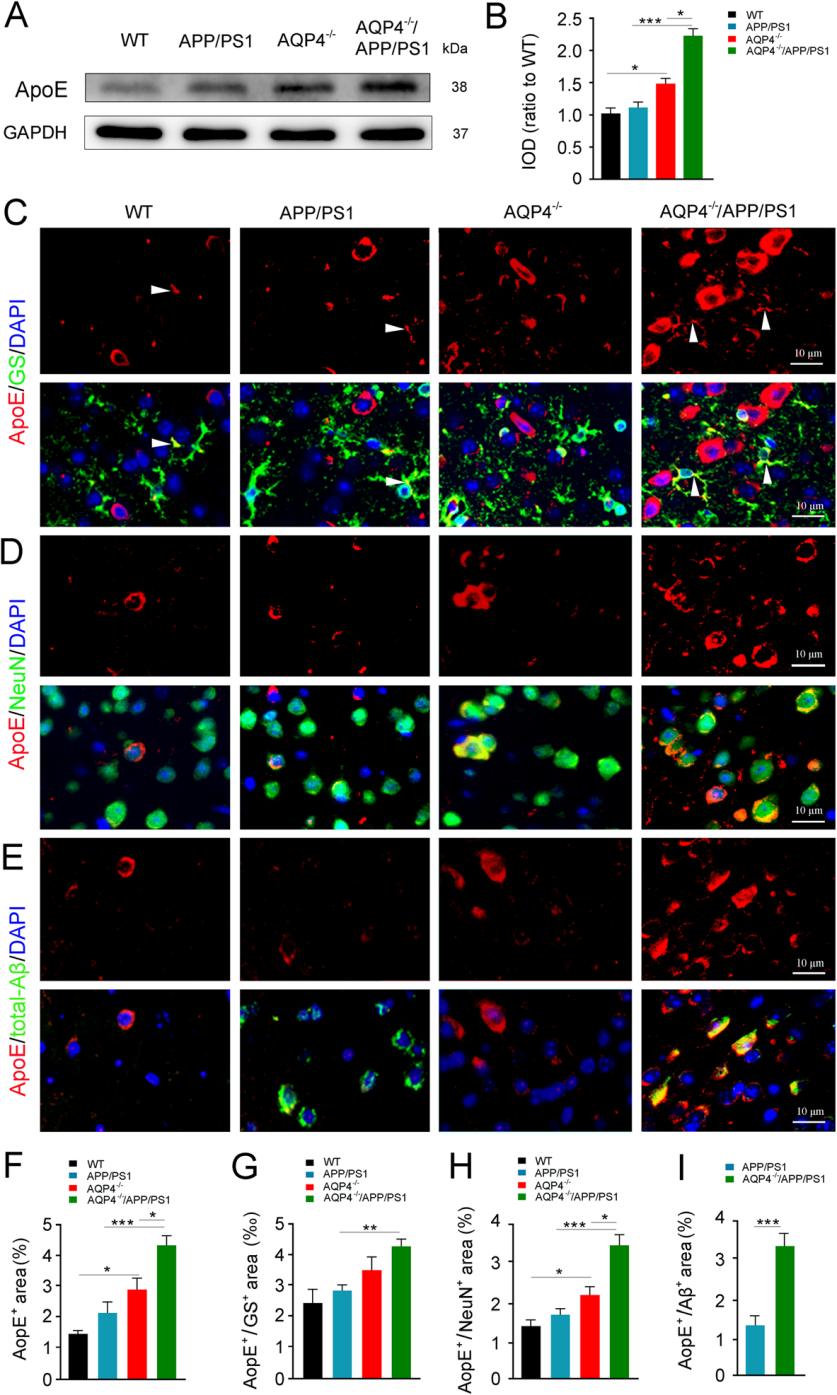
AQP4 deletion increased intraneuronal apoE aggregation in 3-month-old AQP4^−/−^/APP/PS1 mice. **a**, **b** Representative bands of Western blot and densitometry analysis of apoE. **c**–**e** Double immunofluorescence of apoE/GS, apoE/NeuN, and apoE/total-Aβ, respectively. Note that AQP4 deletion in APP/PS1 mice increased apoE immunoreactive intensity in GS-positive astrocytes and NeuN-positive neurons. ApoE and total-Aβ double immunoreactive products were also accumulated. **f**–**i** Quantification of apoE, apoE/GS, apoE/NeuN, and apoE/total-Aβ-positive area fraction in the cerebral cortex, respectively. Data in **i** were analyzed by Student’s *t* test; other data were analyzed by the two-way ANOVA with Newman-Keuls post hoc test. Data are mean ± SEM, *n* = 4 per group, *p < 0.05; **p < 0.01; ***p < 0.001

ApoE, a cholesterol transport protein, also regulates Aβ metabolism, aggregation, and deposition [28]. A previous study reported that the glymphatic system mediates apoE transport within the brain [29]. Therefore, we investigated whether AQP4 deletion affects apoE levels in the brain of 3-month-old APP/PS1 mice. Western blot analysis showed that AQP4 deletion in either WT or APP/PS1 mice resulted in high levels of apoE in the cerebral cortex (*p* < 0.05, *p* < 0.001, respectively; Fig. 4a, b). We further identified localization of apoE within different types of brain cells. Immunofluorescence revealed that the immunoreactive products of apoE in WT mice were localized within the cytoplasm of a small portion of astrocytes (Fig. 4c) and neurons (Fig. 4d). AQP4 deletion in APP/PS1 mice led to increased apoE in astrocytes as well as neurons (Fig. 4c, d, f–h). This result is in agreement with the view that apoE is generated mainly by glial cells, whereas neurons predominantly produce apoE under stressful conditions [30, 31]. Notably, there was a high cellular co-localization of apoE and total-Aβ in AQP4^−/−^/APP/PS1 mice, compared with APP/PS1 mice (*p* < 0.001; Fig. 4e, i). This supports that there is a high affinity between apoE and Aβ, which will facilitate the formation of apoE/Aβ complexes [31–33].

### Knockdown of apoE reduced intraneuronal aggregation of Aβ in the brain of 3-month-old APP/PS1 mice with or without AQP4 deletion

**Fig. 5:**
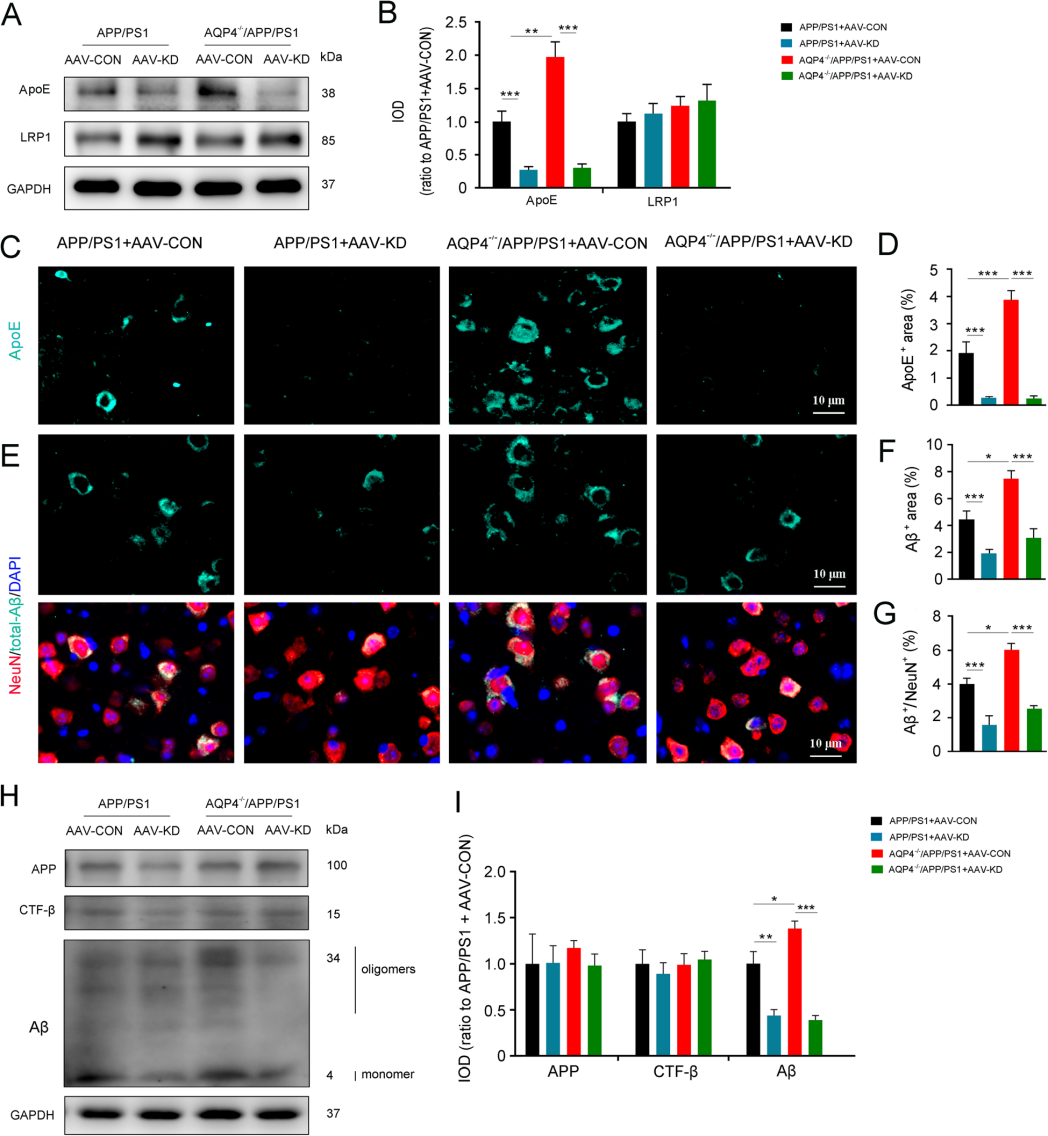
AQP4 deletion increased intraneuronal apoE aggregation in 3-month-old AQP4^−/−^/APP/PS1 mice. **a**, **b** Representative bands of Western blot and densitometry analysis of apoE and LRP1. **c** Immunofluorescence of apoE. ApoE expression was significantly decreased in the cerebral cortex following local injection of AAV encoding apoE siRNAs. **d** Quantification of apoE-positive area fraction in the cerebral cortex. **e** Double immunofluorescence of NeuN and total-Aβ. Total-Aβ expression was also obviously decreased in the cerebral cortex following local injection of AAV encoding apoE siRNAs. **f**, **g** Quantification of total-Aβ and NeuN/total-Aβ-positive area fraction in the cerebral cortex, respectively. **h**, **i** Representative bands of Western blot and densitometry analysis of APP, CTF-β, and Aβ monomers/oligomers in the cortex after local injection of AAV encoding apoE siRNAs. Data were analyzed by two-way ANOVA with Newman-Keuls post hoc test. Data are mean ± SEM, *n* = 4 per group, *p < 0.05; ***p < 0.001

We further determined the effect of downregulating on intraneuronal Aβ load by injection of AAV encoding siRNAs targeting apoE into the frontal cortex (Fig. S5a-d). The apoE levels dramatically decreased in 3-month-old APP/PS1 mice and AQP4^−/−^/APP/PS1 mice that had been pre-injected with the above AAV a month earlier, as revealed by Western blot (Fig. 5a, b) and immunostaining (Fig. 5c, d). As expectedly, local knockdown of apoE decreased intraneuronal accumulation of Aβ in both APP/PS1 mice and AQP4^−/−^/APP/PS1 mice (both *p* < 0.001; Fig. 5e–g). In addition, Western blot results showed that administration of AAV encoding siRNAs targeting apoE significantly decreased the level of Aβ (*p* < 0.05; Fig. 5h, i), without altering APP and CTF-β levels (Fig. 5h, i). These data indicate that dysfunction of the glymphatic system mediated apoE clearance is a main contributor to intraneuronal accumulation of Aβ in the early pathological stages of AD.

## Discussion

**Fig. 6:**
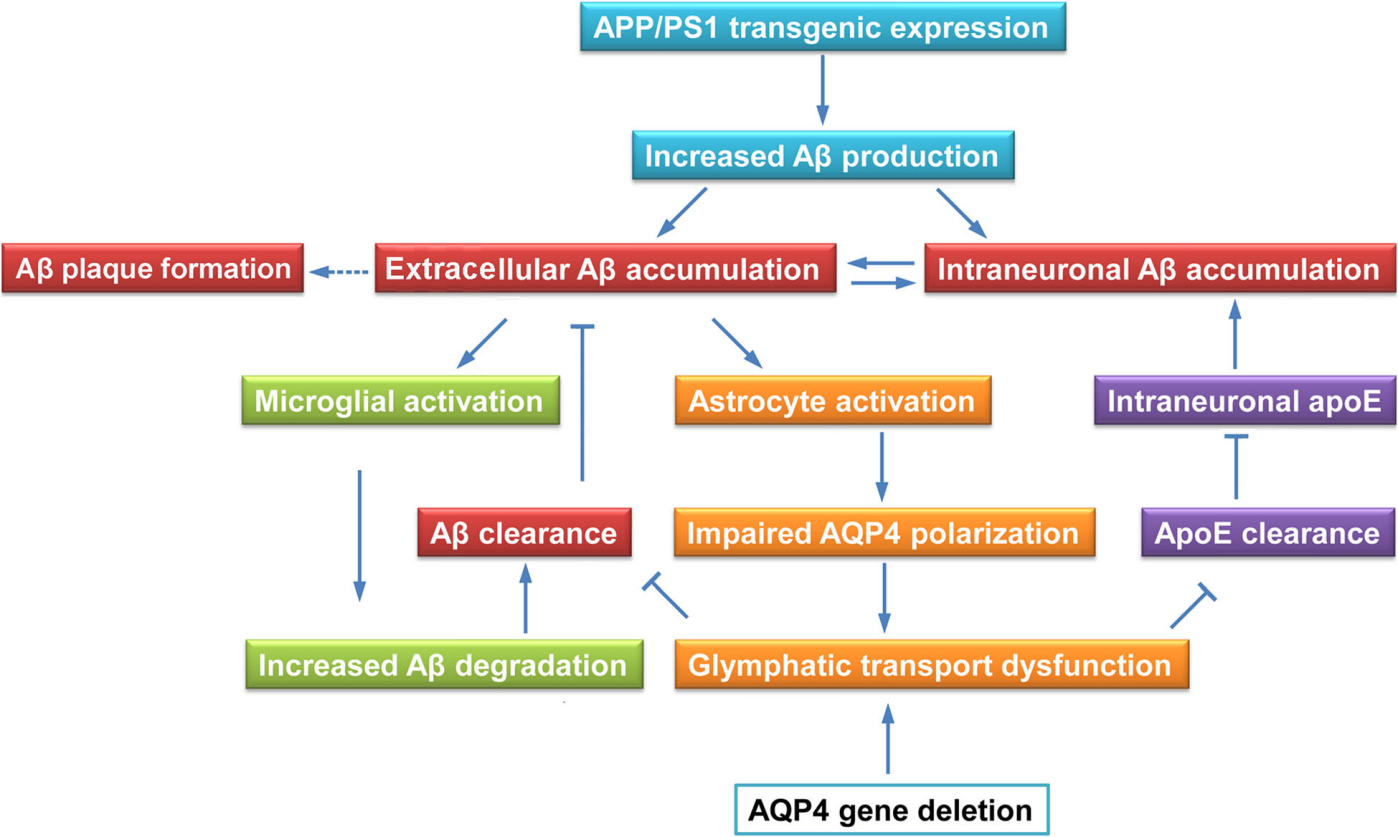
Schematic diagram of our working model for the glymphatic system mediated Aβ and apoE clearance and microglia mediated Aβ degradation synergistically preventing Aβ plague formation in the early stages of the AD mouse model. 3-month-old APP/PS1 mice show extracellular and intraneuronal accumulation of Aβ because of the long-term of expression transgenic APP/PS1. Excessive interstitial Aβ may result in activation of astrocytes with loss of perivascular AQP4 polarization, subsequently impairing glymphatic clearance of Aβ and apoE. On the other hand, microglia undergo activation and promote Aβ clearance, potentially preventing Aβ plaque formation. AQP4 deletion exacerbates glymphatic transport impairment and intraneuronal accumulation of Aβ and apoE, but Aβ plaques have not yet aggregated, which may be related to the increase of phagocytosis of reactive microglia

It is widely accepted that an age-related decline in Aβ clearance is central to the onset of AD, although exact pathogenesis of the disease remains elusive [1, 2]. APP/PS1 mice serve as the most extensively used mouse model of AD [34, 35]. This mouse line exhibits extracellular Aβ deposition until 5–6 months old, mainly attributed to the decompensation of clearance ability under long-term high levels of Aβ burden [36, 37]. Therefore, we chose the 3-month-old time point to characterize the profile of Aβ clearance related markers in the early stage of this AD mouse model (Fig. 6). We further observed the consequences of whole body knockout of the *Aqp4* gene, local microglia elimination, and knockdown of the apoE gene on Aβ load within neurons and extracellular space of the frontal cortex, respectively.

Evidence from recent studies has highlighted that the glymphatic system plays a dominant role in the clearance of ISF Aβ [9–11], and its malfunction may be an aggravating factor in brain aging and AD [11, 12, 38]. The present study has demonstrated that glymphatic transport of a CSF tracer is suppressed in the 3-month-old APP/PS1 brain prior to Aβ plaque deposit. This supports the view that glymphatic dysfunction potentially serves as an early pathological marker of AD [17]. Glymphatic impairment could be associated with mislocalization of AQP4 due to astrocyte activation [10–15]. In addition, previous studies suggested that soluble Aβ peptides, especially Aβ_1–40_, directly inhibit glymphatic transport [17]. However, it remains to be determined which mechanism plays the leading role in suppressing glymphatic function during the early stage of AD.

Evidence from several independent groups has revealed AQP4-dependent glymphatic solute transport in the rodent brain [9, 13, 15], despite others reporting conflicting results [39, 40]. The present study further confirms the importance of AQP4 in glymphatic clearance. Both CSF tracer influx and ISF tracer efflux are decreased following the Aqp4 gene knockout with or without transgenic APP/PS1. Notably, AQP4 deletion dramatically reduces the transport rate of intrastriatal TR-d3 into the dcLNs. This result further emphasizes that AQP4-mediated rapid water transport is involved in glymphatic macromolecule drainage from the brain into the peripheral lymphatic system.

The current results have revealed that Aβ is aggregated within frontal cortex neurons of APP/PS1 mice at 3 months old, compared to WT controls, but has yet to cause obvious Aβ plaque formation. The absence of AQP4 does not cause Aβ plaque formation, but rather exacerbates intraneuronal Aβ aggregation in the early stage of this AD mouse model. This result supports the view that build-up of Aβ within neurons may play a crucial role in plaque formation [41–43].

The following compensatory mechanisms related Aβ clearance might contribute to no Aβ plaque formation despite an intensified glymphatic clearance dysfunction caused by AQP4 deletion. First, there is normal expression level of LRP1, which is responsible for transfer of Aβ outside the brain [6, 7]. Second, dural lymphatic drainage remains intact. Recent studies have shown that brain interstitial fluid and macromolecules can be transported into the dcLNs through meningeal lymphatics [44–46]. Our previous studies have also suggested that the extra cranial brain clearance pathway remains intact in 6-month-old APP/PS1 mice, but the deposition of Aβ plaques significantly increases after the mice received bilateral ligation of afferent vessels of the dcLNs [47]. More importantly, clearance of Aβ may be improved by activation of microglia [24, 25]. In the present study, Aβ-positive products, and CD68, a phagocyte-associated marker [22], are detected in cell bodies of activated microglia in the frontal cortex of AQP4^−/−^/APP/PS1 mice. Furthermore, Lamp1/Aβ/Iba1 triple immunostaining has revealed that a considerable portion of Aβ-positive products are located within lysosomes of AQP4^−/−^/APP/PS1 microglia. In addition, there was increased activity of IDE, mainly produced by microglia within the brain [23], in the cerebral cortex of AQP4^−/−^/APP/PS1 mice. Together, these results indicate that microglial phagocytosis and enzymatic degradation are potentially beneficial to prevent accumulation of extracellular Aβ. Indeed, clodronate liposomes that eliminates microglia for only 5 days cause Aβ plaque deposition in the frontal cortex of 3-month-old AQP4^−/−^/APP/PS1 mice.

Interestingly, eliminating microglia does not enhance Aβ plaque formation in 3-month-old APP/PS1 mice, which could be attributed to an alleviated impairment of glymphatic transport compared to that in AQP4^−/−^/APP/PS1 mice with the same age. In addition, we have discovered a significant reduction of LRP1, and more severe Aβ plaque deposition, in the brain parenchyma and vascular amyloidosis of 12-month-old AQP4^−/−^/APP/PS1 mice, compared with their APP/PS1 littermates [19]. These results suggest that glial cell phagocytosis, enzymatic degradation, glymphatic clearance, and LRP1-mediated transport working together prevent Aβ plaque formation. However, the compensatory roles of these Aβ clearance systems are gradually diminished, resulting in extracellular Aβ aggregation with pathological progression of AD.

Our experimental results have illustrated that the absence of AQP4 does not increase reactive astroglosis in cerebral cortex of 3-month-old APP/PS1 mice, which is contrary to results found in microglia. Our previous study demonstrated that 6-month AQP4^−/−^/APP/PS1 mice have more extensive reactive gliosis than APP/PS1 mice, and at 12 months a large number of astrocytes around Aβ plaques undergo atrophy [19]. An early study from our laboratory has reported that AQP4 deficiency decreases Aβ uptake in cultured astrocytes, and in turn attenuates changes in mitogen-activated protein kinase pathways, finally reducing astrocyte activity [48]. Similarly, reduced reactive astroglosis is likewise observed in 7–9-month-old AQP4^−/−^/5xTg-AD mouse brains [49]. These data together indicate that long-term loss of AQP4 not only damages glymphatic clearance of Aβ, but also decreases astrocyte phagocytosis of Aβ, in turn exacerbating the pathological progression of AD.

ApoE, a lipid carrier protein, is produced by astrocytes and secreted as high-density lipoprotein-like particles in the brain. ApoE can directly bind to Aβ amino-acid residues [12–28], subsequently forming apoE/Aβ complex within neurons and extracellular space [31–33]. Co-deposition of apoE with Aβ is a key step for Aβ fibrillization and plaque formation [50, 51]. Therefore, blocking apoE/Aβ interaction may be a promising strategy for improving clearance of soluble Aβ from the brain, and preventing the onset of AD. However, there is still lack of safe and effective routes to inhibit apoE aggregation within the brain parenchyma. The current study has revealed Aβ and apoE co-accumulation within neurons prior to plague formation in 3-month-old APP/PS1 mice. A recent study has reported that the glymphatic system contributes to the delivery of CSF-derived apoE to neurons. CSF apoE distribution in brain is reduced in AQP4^−/−^ mice [17]. Consistently, we have shown that AQP4 deletion increases Aβ and apoE co-aggregation within neurons, supporting that glymphatic dysfunction is an important factor in Aβ pathogenesis. Moreover, the current results suggest that knockdown of apoE expression significantly attenuates intraneuronal aggregation of Aβ. This is consistent with findings reporting the mitigating effects of apoE knock-out on fibrillar Aβ deposits and neurodegeneration in several AD-Tg mouse models [52–55].

In addition, accumulated evidence has highlighted that inheritance of the APOE ε4 allele is the strongest known risk factor for sporadic AD [56, 57]. A previous study has indicated that both apoE2, apoE3, and apoE4 isoforms contribute to Aβ deposition in APP/PS1 mice [58]. A recent study has revealed that apoE secreted by glia stimulates neuronal Aβ production with an ApoE4 > ApoE3 > ApoE2 potency rank order [59]. Further study is necessary to identify which subtype apoE is affected by glymphatic dysfunction in this AD model, due to the fact that a facilitating effect of glymphatic transport of CSF-derived apoE is in an isoform-specific manner (apoE2 > apoE3 > apoE4) [17]. It is necessary to further determine whether increased ApoE production is also involved in accumulation of apoE in the brain after deletion of AQP4. Exploring this issue is helpful to reveal the interaction between AQP4 and apoE in the pathogenesis of AD.

Apart from Aβ, evidence from recent studies suggests that apoE influencing tau pathology and tau-mediated neurodegeneration is also in an isoform-dependent manner [60, 61]. Furthermore, apoE ε4 accumulation impairs glial responsiveness, lipid transport, and cerebrovascular integrity and function, which might be independent of Aβ-related pathways [31, 62, 63]. These results indicate that promoting glymphatic clearance of macromolecular metabolic proteins including Aβ, Tau, and apoE may counteract pathological cascades of AD in multiple aspects, potentially delaying the onset and development of the disease.

## Conclusion

The present study has characterized pathological features of Aβ clearance systems of 3-month-old APP/PS1 mice. AQP4 deletion exacerbates glymphatic clearance impairment with increases in intracellular accumulation of Aβ and apoE in APP/PS1 brain. Furthermore, it also improves IDE activity and microglia phagocytosis without aggravating Aβ plaque deposition and spatial cognitive dysfunction. Eliminating microglial cells causes Aβ deposition in the brain of AQP4^−/−^/APP/PS1 mice, but not APP/PS1 mice. Knockdown of apoE reduce intraneuronal Aβ levels in APP/PS1 mice with or without AQP4. These results have preliminarily revealed an interactive compensation between glymphatic clearance and microglial phagocytosis in inhibiting brain Aβ accumulation in the early stage of AD-like progression. This might provide a promising strategy for preventing the onset of AD.

## Supplementary information

**Additional file 1.** Morris water maze. The Morris water maze test was conducted to measure mouse long-term spatial cognitive function, as described previously [19]. Four Training was performed over 7 consecutive days, with 4 trials per day. During the first two days, mice were trained to find a dark-colored cylindrical platform with a diameter of 10 cm, sitting 0.5 cm above the water surface. Mice did not receive the next hidden platform tests if they had apparent motor and/or visual deficits indicated by low swimming speed (< 75 mm/s) or long escape latency (> 50 s). On the 3rd day, the platform was submerged 1 cm below the surface of the water and moved to the opposite quadrant. Escape latency, swimming distance and swimming speed were calculated. On day 8, the hidden platform was removed, allowing mice to swim freely in the pool for 60 s. The percentage of time spent in the target quadrant and the number of crossing where the platform had been previously located were analyzed. Y-maze test. The Y-maze test was performed to evaluate mouse short-term spatial working memory, as previously described [19]. One arm, termed the novel arm, was blocked by a black baffle, allowing the mice to only move in the other two arms for 5 min. Two hours later, the novel arm was opened, allowing mice to freely move throughout the three arms. The percentage of time traveled in, number of entries into the novel arm, as well as traveling speed during the test, was calculated. Mouse activity in the aforementioned behavioral apparatuses was collected by a digital video camera connected to a computer-controlled system (Beijing Sunny Instruments Co. Ltd., China). All tests were performed by two independent experimenters, who were each blind to the treatment schedule.

**Additional file 2: Fig. S1.** No obvious effects of AQP4 deletion on spatial cognitive function of 3-month-old APP/PS1 mice. a, b The mean escape latency and swimming speed during the training period of the Morris water maze test. c The number of crossing the platform. d The percentage of time in the target quadrant. e The number of entries into the novel arm. f The percentage of time in the novel arm in the Y-maze. Data in S1a, b were analyzed by repeated-measures ANOVA with post hoc Student-Newman-Keuls test. Other Data were analyzed by ANOVA with post hoc Student Newman-Keuls test. Data are means ± SEM. *n* = 12 per group. **Fig. S2.** AQP4 deletion did not affect astrocyte activation in 3-month-old APP/PS1 mice. a, b Double immunofluorescence and quantification for GFAP and total-Aβ in the cortex. c, d Immunofluorescence and quantification for GS positive astrocytes in the cortex of APP/PS1 mice and AQP4^−/−^/APP/PS1 mice. Data are means ± SEM. *n* = 4 per group, two-way ANOVA with Newman-Keuls post-hoc test. **Fig. S3.** AQP4 polarization was impaired in the cerebral cortex of 3-month-old APP/PS1 mice. a Double immunofluorescence for AQP4 and GFAP. b Quantitative analyses of the AQP4 polarization. Data are means ± SEM. n = 4 per group, Student’s t-test. **p < 0.01; ***p < 0.001. **Fig. S4.** Increased astrocyte activation in the cortex in 3-month-old APP/PS1 mice and AQP4^−/−^/APP/PS1 mice receiving local injection of clodronate liposomes. a, b Double immunofluorescence and quantification for total-Aβ and GFAP in the cortex. Data are means ± SEM. n = 4 per group, two-way ANOVA with Newman-Keuls post-hoc test. **Fig. S5.** An image shows GFP expression in the cortex one month after injection of AAV encoding apoE siRNAs. a GFP positive area represented where the AAVs was injected. b-d Double immunofluorescence for GFP and GFAP. Note that apoE siRNAs were expressed in GFAP positive astrocytes (arrowheads).

## Data Availability

The datasets used and/or analyzed during the current study are available from the corresponding author on reasonable request.

## Abbreviations

AD: Alzheimer’s disease
AAV: Adeno-associated virus
apoE: Apolipoprotein E
APP: Amyloid precursor protein
AQP4: Aquaporin 4
Aβ: Beta-amyloid
BACE1: β-site amyloid precursor protein-cleaving enzyme 1
CSF: Cerebral spinal fluid
dcLNs: Deep cervical lymph nodes
CTF-β: β-C-Terminal fragment
GFAP: Glial fibrillary acidic protein
GS: Glutamine synthetase
Iba1: Ionized calcium binding adapter molecule 1
IDE: Insulin-degrading enzyme
ISF: Interstitial fluid
Lamp1: Lysosome-associated membrane glycoprotein 1
LRP1: Low-density lipoprotein receptor-related proteins 1
NEP: Neprilysin
PFA: Paraformaldehyde
PS1: Presenilin-1
WT: Wild type
