# Supplementary material for: Microglia prevent beta-amyloid plaque formation in the early stage of an Alzheimer’s disease mouse model with suppression of glymphatic clearance
Source: Alzheimers Res Ther. 2020 Oct 2;12:125. doi: 10.1186/s13195-020-00688-1 (PMC7532614; doi:10.1186/s13195-020-00688-1)
Supplement: Supplementary file 1 — Additional file 1. Morris water maze. The Morris water maze test was conducted to measure mouse long-term spatial cognitive function, as described previously [19]. Four Training was performed over 7 consecutive days, with 4 trials per day. During the first two days, mice were trained to find a dark-colored cylindrical platform with a diameter of 10 cm, sitting 0.5 cm above the water surface. Mice did not receive the next hidden platform tests if they had apparent motor and/or visual deficits indicated by low swimming speed (< 75 mm/s) or long escape latency (> 50 s). On the 3rd day, the platform was submerged 1 cm below the surface of the water and moved to the opposite quadrant. Escape latency, swimming distance and swimming speed were calculated. On day 8, the hidden platform was removed, allowing mice to swim freely in the pool for 60 s. The percentage of time spent in the target quadrant and the number of crossing where the platform had been previously located were analyzed. Y-maze test. The Y-maze test was performed to evaluate mouse short-term spatial working memory, as previously described [19]. One arm, termed the novel arm, was blocked by a black baffle, allowing the mice to only move in the other two arms for 5 min. Two hours later, the novel arm was opened, allowing mice to freely move throughout the three arms. The percentage of time traveled in, number of entries into the novel arm, as well as traveling speed during the test, was calculated. Mouse activity in the aforementioned behavioral apparatuses was collected by a digital video camera connected to a computer-controlled system (Beijing Sunny Instruments Co. Ltd., China). All tests were performed by two independent experimenters, who were each blind to the treatment schedule. [file 13195_2020_688_MOESM1_ESM.docx]

**Supplementary materials**

**Additional file 1.**

**Morris water maze**

The Morris water maze test was conducted to measure mouse long-term spatial cognitive function, as described previously [19]. Four Training was performed over 7 consecutive days, with 4 trials per day. During the first two days, mice were trained to find a dark-colored cylindrical platform with a diameter of 10 cm, sitting 0.5 cm above the water surface. Mice did not receive the next hidden platform tests if they had apparent motor and/or visual deficits indicated by low swimming speed (< 75 mm/s) or long escape latency (> 50 s). On the 3rd day, the platform was submerged 1 cm below the surface of the water and moved to the opposite quadrant. Escape latency, swimming distance and swimming speed were calculated. On day 8, the hidden platform was removed, allowing mice to swim freely in the pool for 60 s. The percentage of time spent in the target quadrant and the number of crossing where the platform had been previously located were analyzed.

**Y-maze test**

The Y-maze test was performed to evaluate mouse short-term spatial working memory, as previously described [19]. One arm, termed the novel arm, was blocked by a black baffle, allowing the mice to only move in the other two arms for 5 min. Two hours later, the novel arm was opened, allowing mice to freely move throughout the three arms. The percentage of time traveled in, number of entries into the novel arm, as well as travelling speed during the test, was calculated.

Mouse activity in the aforementioned behavioral apparatuses was collected by a digital video camera connected to a computer-controlled system (Beijing Sunny Instruments Co. Ltd, China). All tests were performed by two independent experimenters, who were each blind to the treatment schedule.
