# Supplementary material for: Microglia prevent beta-amyloid plaque formation in the early stage of an Alzheimer’s disease mouse model with suppression of glymphatic clearance
Source: Alzheimers Res Ther. 2020 Oct 2;12:125. doi: 10.1186/s13195-020-00688-1 (PMC7532614; doi:10.1186/s13195-020-00688-1)
Supplement: Supplementary file 2 — Additional file 2: Fig. S1. No obvious effects of AQP4 deletion on spatial cognitive function of 3-month-old APP/PS1 mice. a, b The mean escape latency and swimming speed during the training period of the Morris water maze test. c The number of crossing the platform. d The percentage of time in the target quadrant. e The number of entries into the novel arm. f The percentage of time in the novel arm in the Y-maze. Data in S1a, b were analyzed by repeated-measures ANOVA with post hoc Student-Newman-Keuls test. Other Data were analyzed by ANOVA with post hoc Student Newman-Keuls test. Data are means ± SEM. n = 12 per group. Fig. S2. AQP4 deletion did not affect astrocyte activation in 3-month-old APP/PS1 mice. a, b Double immunofluorescence and quantification for GFAP and total-Aβ in the cortex. c, d Immunofluorescence and quantification for GS positive astrocytes in the cortex of APP/PS1 mice and AQP4−/−/APP/PS1 mice. Data are means ± SEM. n = 4 per group, two-way ANOVA with Newman-Keuls post-hoc test. Fig. S3. AQP4 polarization was impaired in the cerebral cortex of 3-month-old APP/PS1 mice. a Double immunofluorescence for AQP4 and GFAP. b Quantitative analyses of the AQP4 polarization. Data are means ± SEM. n = 4 per group, Student’s t-test. **p < 0.01; ***p < 0.001. Fig. S4. Increased astrocyte activation in the cortex in 3-month-old APP/PS1 mice and AQP4−/−/APP/PS1 mice receiving local injection of clodronate liposomes. a, b Double immunofluorescence and quantification for total-Aβ and GFAP in the cortex. Data are means ± SEM. n = 4 per group, two-way ANOVA with Newman-Keuls post-hoc test. Fig. S5. An image shows GFP expression in the cortex one month after injection of AAV encoding apoE siRNAs. a GFP positive area represented where the AAVs was injected. b-d Double immunofluorescence for GFP and GFAP. Note that apoE siRNAs were expressed in GFAP positive astrocytes (arrowheads). [file 13195_2020_688_MOESM2_ESM.zip › Additional file 2.docx]

**Additional file 2.**

**Figure S1.** No obvious effects of AQP4 deletion on spatial cognitive function of 3-month-old APP/PS1 mice. **a, b** The mean escape latency and swimming speed during the hidden platform training period of the Morris water maze test. **c** The number of crossing the platform area. **d** The percentage of time spent in the target quadrant. **e** The number of entries into the novel arm. **f** The percentage of time spent in the novel arm in the Y-maze. Data in **S1a**, **b** were analyzed by repeated-measures ANOVA with post hoc Student-Newman-Keuls test. Other Data were analyzed by ANOVA with post hoc Student Newman-Keuls test. Data are means ± SEM. n = 12 per group.

**Figure S2.** AQP4 deletion did not affect astrocyte activation in 3-month-old APP/PS1 mice. **a** Double immunofluorescence for GFAP and total-Aβ. **b** Quantification of GFAP positive area in the cerebral cortex. **c** Immunofluorescence for GS positive astrocytes in the cortex. **d** Quantification of GS positive area in the cerebral cortex. GS or GFAP positive astrocytes were mildly activated in the cerebral cortex of APP/PS1 mice and AQP4^-/-^/APP/PS1 mice. Data are means ± SEM. n = 4 per group, two-way ANOVA with Newman-Keuls post-hoc test.

**Figure S3.** AQP4 polarization was impaired in the cerebral cortex of 3-month-old APP/PS1 mice. **a** Double immunofluorescence for AQP4 and GFAP. In WT mice, AQP4 was specifically expressed abutting the microvessels (arrow) and pia surface (arrowhead). By contrast, AQP4 immunoreactivity was abnormally increased in the adjacent parenchyma (indicated by stars) in APP/PS1 mice. There was no immunoreactive signal for AQP4 in the cortex of AQP4^-/-^ mice and AQP4^-/-^/APP/PS1 mice. **b** Quantitative analyses of the AQP4 polarization abutting pia mater and microvessels. Data are means ± SEM. n = 4 per group, Student’s t-test. ***p* < 0.01; ****p* < 0.001.

**Figure S4.** Increased astrocyte activation in the frontal cortex in 3-month-old APP/PS1 mice and AQP4^-/-^/APP/PS1 mice receiving local injection of clodronate liposomes. **a** Double immunofluorescence for total-Aβ and GFAP. GFAP positive astrocytes further underwent activation both in APP/PS1 mice and AQP4^-/-^/APP/PS1 received clodronate liposome treatment. **b** Quantification of GFAP positive area fraction in the cerebral cortex. Data are means ± SEM. n = 4 per group, two-way ANOVA with Newman-Keuls post-hoc test.

**Figure S5.** An image shows GFP expression in the frontal cortex one month after injection of AAV encoding apoE siRNAs. **a** GFP positive area represented where the AAVs was injected and apoE siRNAs were expressed. **b-d** Double immunofluorescence for GFP and GFAP. Note that apoE siRNAs were expressed in GFAP positive astrocytes (arrowheads).
