# Supplementary figures and images for: Microglia prevent beta-amyloid plaque formation in the early stage of an Alzheimer’s disease mouse model with suppression of glymphatic clearance
Source: Alzheimers Res Ther. 2020 Oct 2;12:125. doi: 10.1186/s13195-020-00688-1 (PMC7532614; doi:10.1186/s13195-020-00688-1)

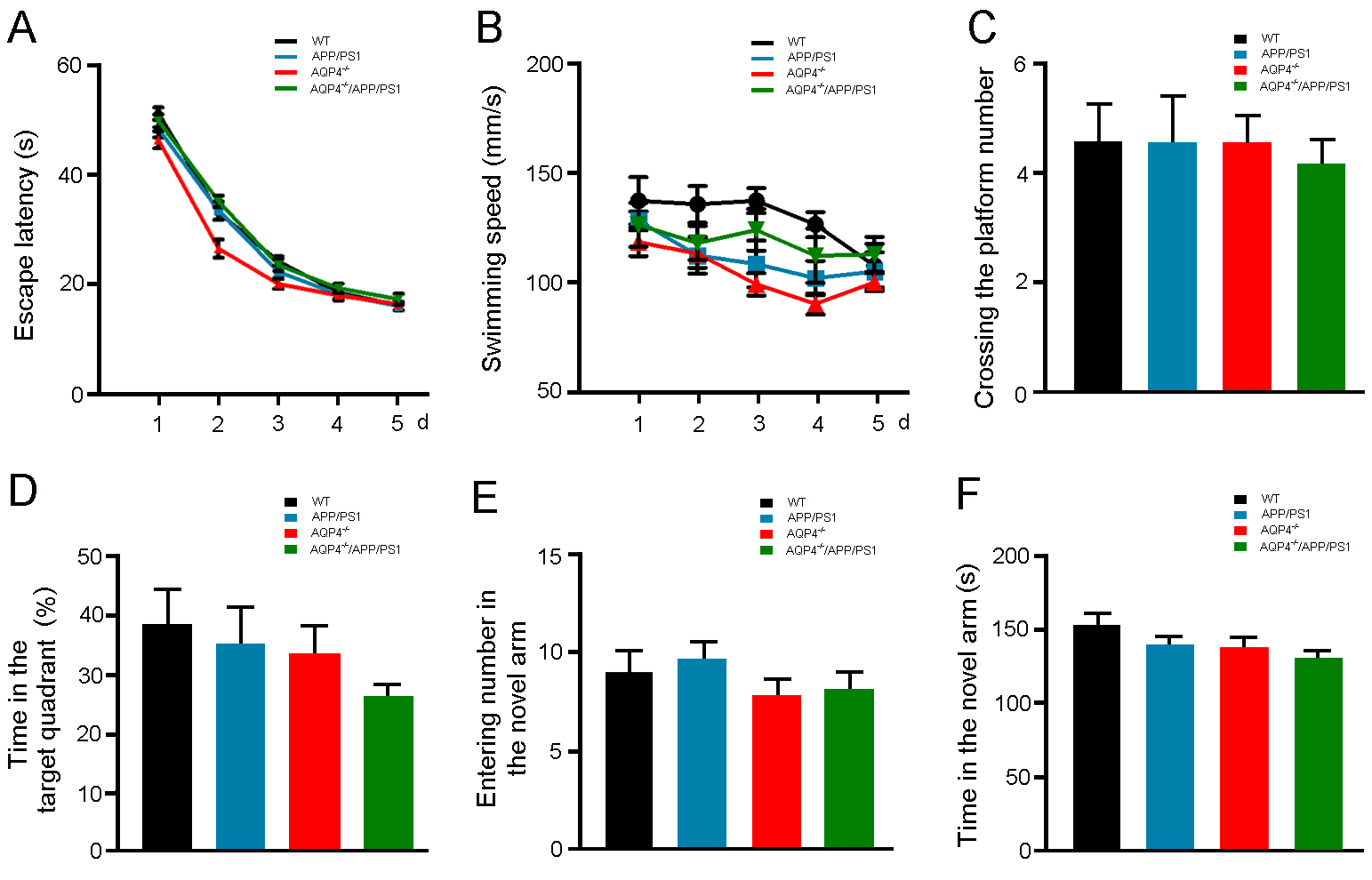

Supplement: Supplementary file 2 — Additional file 2: Fig. S1. No obvious effects of AQP4 deletion on spatial cognitive function of 3-month-old APP/PS1 mice. a, b The mean escape latency and swimming speed during the training period of the Morris water maze test. c The number of crossing the platform. d The percentage of time in the target quadrant. e The number of entries into the novel arm. f The percentage of time in the novel arm in the Y-maze. Data in S1a, b were analyzed by repeated-measures ANOVA with post hoc Student-Newman-Keuls test. Other Data were analyzed by ANOVA with post hoc Student Newman-Keuls test. Data are means ± SEM. n = 12 per group. Fig. S2. AQP4 deletion did not affect astrocyte activation in 3-month-old APP/PS1 mice. a, b Double immunofluorescence and quantification for GFAP and total-Aβ in the cortex. c, d Immunofluorescence and quantification for GS positive astrocytes in the cortex of APP/PS1 mice and AQP4−/−/APP/PS1 mice. Data are means ± SEM. n = 4 per group, two-way ANOVA with Newman-Keuls post-hoc test. Fig. S3. AQP4 polarization was impaired in the cerebral cortex of 3-month-old APP/PS1 mice. a Double immunofluorescence for AQP4 and GFAP. b Quantitative analyses of the AQP4 polarization. Data are means ± SEM. n = 4 per group, Student’s t-test. **p < 0.01; ***p < 0.001. Fig. S4. Increased astrocyte activation in the cortex in 3-month-old APP/PS1 mice and AQP4−/−/APP/PS1 mice receiving local injection of clodronate liposomes. a, b Double immunofluorescence and quantification for total-Aβ and GFAP in the cortex. Data are means ± SEM. n = 4 per group, two-way ANOVA with Newman-Keuls post-hoc test. Fig. S5. An image shows GFP expression in the cortex one month after injection of AAV encoding apoE siRNAs. a GFP positive area represented where the AAVs was injected. b-d Double immunofluorescence for GFP and GFAP. Note that apoE siRNAs were expressed in GFAP positive astrocytes (arrowheads). [file 13195_2020_688_MOESM2_ESM.zip › Figure S1.tif]

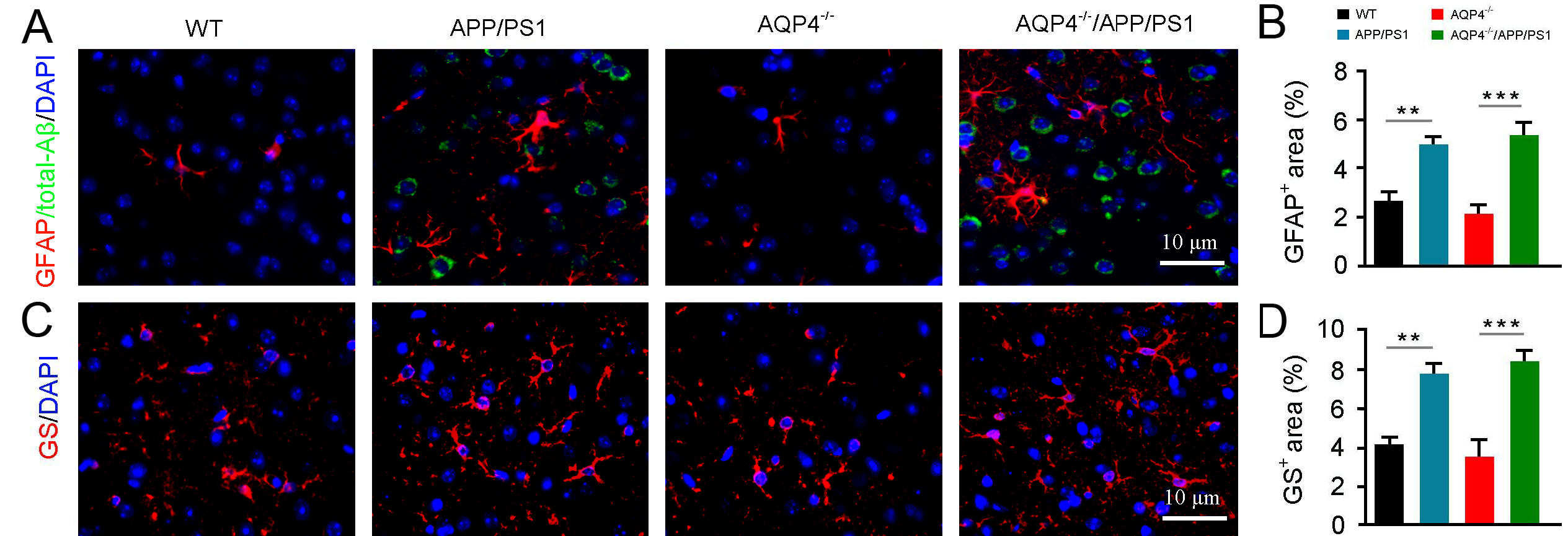

Supplement: Supplementary file 2 — Additional file 2: Fig. S1. No obvious effects of AQP4 deletion on spatial cognitive function of 3-month-old APP/PS1 mice. a, b The mean escape latency and swimming speed during the training period of the Morris water maze test. c The number of crossing the platform. d The percentage of time in the target quadrant. e The number of entries into the novel arm. f The percentage of time in the novel arm in the Y-maze. Data in S1a, b were analyzed by repeated-measures ANOVA with post hoc Student-Newman-Keuls test. Other Data were analyzed by ANOVA with post hoc Student Newman-Keuls test. Data are means ± SEM. n = 12 per group. Fig. S2. AQP4 deletion did not affect astrocyte activation in 3-month-old APP/PS1 mice. a, b Double immunofluorescence and quantification for GFAP and total-Aβ in the cortex. c, d Immunofluorescence and quantification for GS positive astrocytes in the cortex of APP/PS1 mice and AQP4−/−/APP/PS1 mice. Data are means ± SEM. n = 4 per group, two-way ANOVA with Newman-Keuls post-hoc test. Fig. S3. AQP4 polarization was impaired in the cerebral cortex of 3-month-old APP/PS1 mice. a Double immunofluorescence for AQP4 and GFAP. b Quantitative analyses of the AQP4 polarization. Data are means ± SEM. n = 4 per group, Student’s t-test. **p < 0.01; ***p < 0.001. Fig. S4. Increased astrocyte activation in the cortex in 3-month-old APP/PS1 mice and AQP4−/−/APP/PS1 mice receiving local injection of clodronate liposomes. a, b Double immunofluorescence and quantification for total-Aβ and GFAP in the cortex. Data are means ± SEM. n = 4 per group, two-way ANOVA with Newman-Keuls post-hoc test. Fig. S5. An image shows GFP expression in the cortex one month after injection of AAV encoding apoE siRNAs. a GFP positive area represented where the AAVs was injected. b-d Double immunofluorescence for GFP and GFAP. Note that apoE siRNAs were expressed in GFAP positive astrocytes (arrowheads). [file 13195_2020_688_MOESM2_ESM.zip › Figure S2.tif]

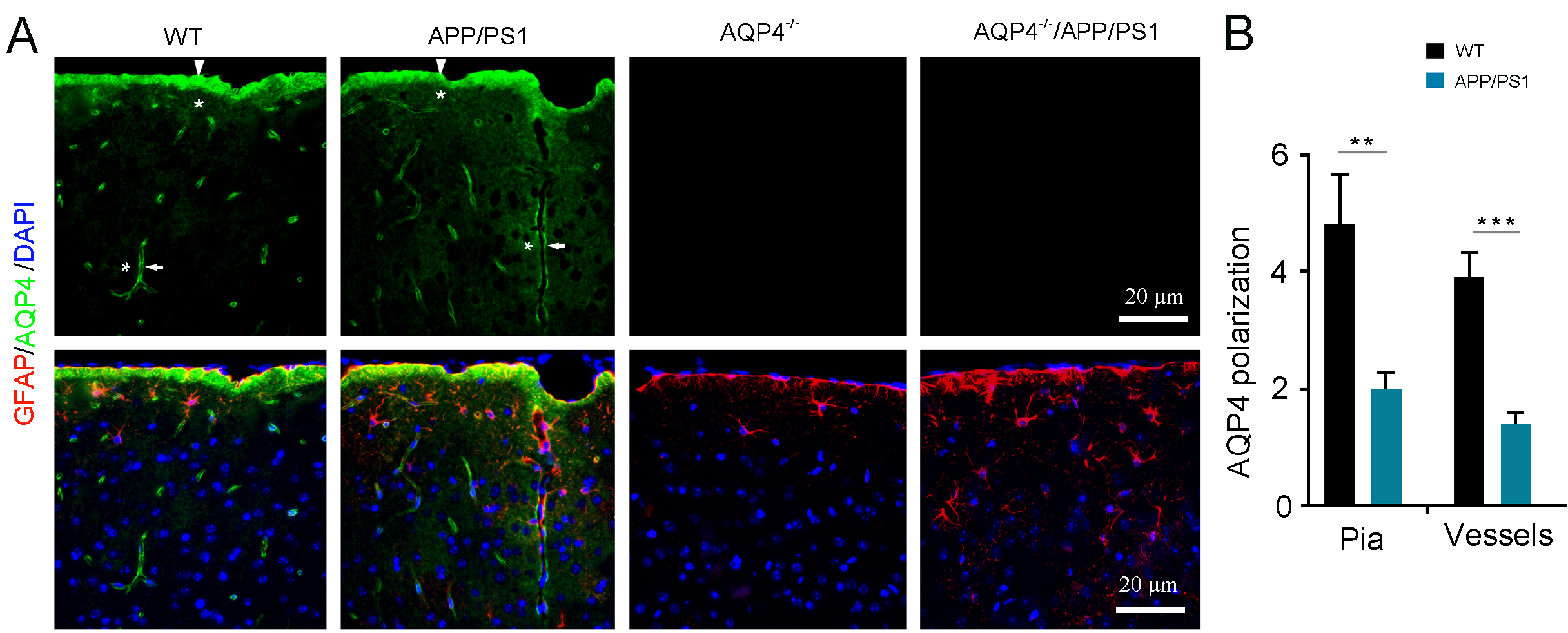

Supplement: Supplementary file 2 — Additional file 2: Fig. S1. No obvious effects of AQP4 deletion on spatial cognitive function of 3-month-old APP/PS1 mice. a, b The mean escape latency and swimming speed during the training period of the Morris water maze test. c The number of crossing the platform. d The percentage of time in the target quadrant. e The number of entries into the novel arm. f The percentage of time in the novel arm in the Y-maze. Data in S1a, b were analyzed by repeated-measures ANOVA with post hoc Student-Newman-Keuls test. Other Data were analyzed by ANOVA with post hoc Student Newman-Keuls test. Data are means ± SEM. n = 12 per group. Fig. S2. AQP4 deletion did not affect astrocyte activation in 3-month-old APP/PS1 mice. a, b Double immunofluorescence and quantification for GFAP and total-Aβ in the cortex. c, d Immunofluorescence and quantification for GS positive astrocytes in the cortex of APP/PS1 mice and AQP4−/−/APP/PS1 mice. Data are means ± SEM. n = 4 per group, two-way ANOVA with Newman-Keuls post-hoc test. Fig. S3. AQP4 polarization was impaired in the cerebral cortex of 3-month-old APP/PS1 mice. a Double immunofluorescence for AQP4 and GFAP. b Quantitative analyses of the AQP4 polarization. Data are means ± SEM. n = 4 per group, Student’s t-test. **p < 0.01; ***p < 0.001. Fig. S4. Increased astrocyte activation in the cortex in 3-month-old APP/PS1 mice and AQP4−/−/APP/PS1 mice receiving local injection of clodronate liposomes. a, b Double immunofluorescence and quantification for total-Aβ and GFAP in the cortex. Data are means ± SEM. n = 4 per group, two-way ANOVA with Newman-Keuls post-hoc test. Fig. S5. An image shows GFP expression in the cortex one month after injection of AAV encoding apoE siRNAs. a GFP positive area represented where the AAVs was injected. b-d Double immunofluorescence for GFP and GFAP. Note that apoE siRNAs were expressed in GFAP positive astrocytes (arrowheads). [file 13195_2020_688_MOESM2_ESM.zip › Figure S3.tif]

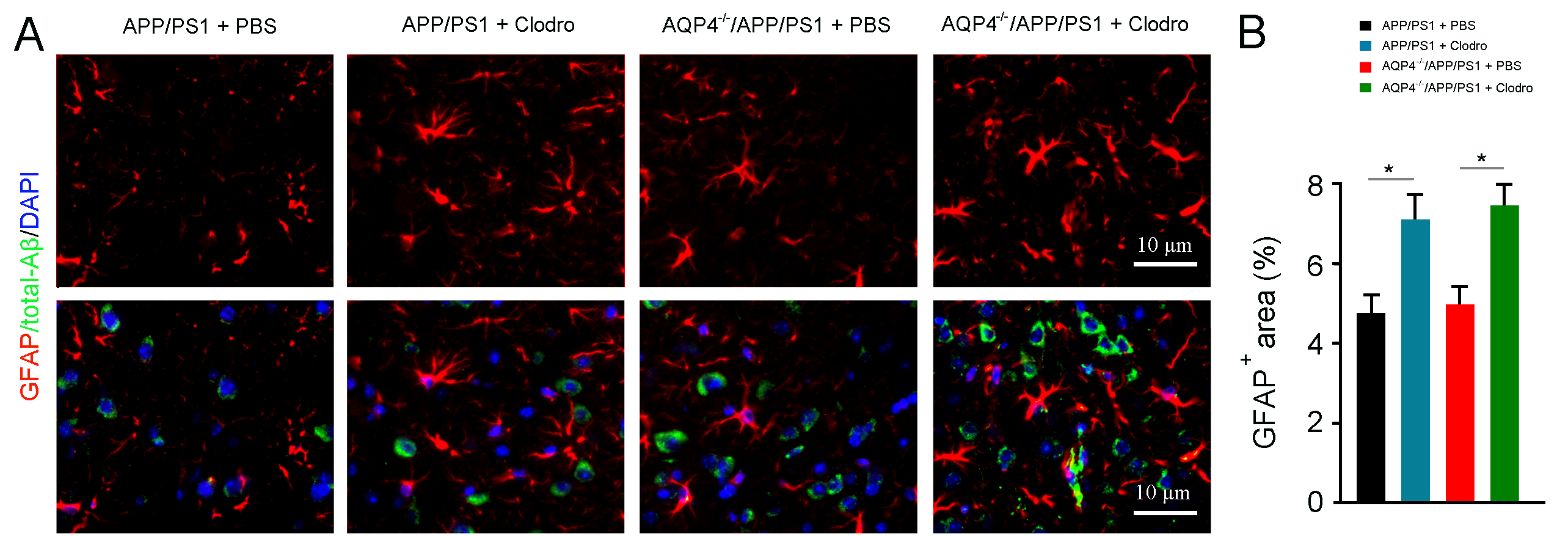

Supplement: Supplementary file 2 — Additional file 2: Fig. S1. No obvious effects of AQP4 deletion on spatial cognitive function of 3-month-old APP/PS1 mice. a, b The mean escape latency and swimming speed during the training period of the Morris water maze test. c The number of crossing the platform. d The percentage of time in the target quadrant. e The number of entries into the novel arm. f The percentage of time in the novel arm in the Y-maze. Data in S1a, b were analyzed by repeated-measures ANOVA with post hoc Student-Newman-Keuls test. Other Data were analyzed by ANOVA with post hoc Student Newman-Keuls test. Data are means ± SEM. n = 12 per group. Fig. S2. AQP4 deletion did not affect astrocyte activation in 3-month-old APP/PS1 mice. a, b Double immunofluorescence and quantification for GFAP and total-Aβ in the cortex. c, d Immunofluorescence and quantification for GS positive astrocytes in the cortex of APP/PS1 mice and AQP4−/−/APP/PS1 mice. Data are means ± SEM. n = 4 per group, two-way ANOVA with Newman-Keuls post-hoc test. Fig. S3. AQP4 polarization was impaired in the cerebral cortex of 3-month-old APP/PS1 mice. a Double immunofluorescence for AQP4 and GFAP. b Quantitative analyses of the AQP4 polarization. Data are means ± SEM. n = 4 per group, Student’s t-test. **p < 0.01; ***p < 0.001. Fig. S4. Increased astrocyte activation in the cortex in 3-month-old APP/PS1 mice and AQP4−/−/APP/PS1 mice receiving local injection of clodronate liposomes. a, b Double immunofluorescence and quantification for total-Aβ and GFAP in the cortex. Data are means ± SEM. n = 4 per group, two-way ANOVA with Newman-Keuls post-hoc test. Fig. S5. An image shows GFP expression in the cortex one month after injection of AAV encoding apoE siRNAs. a GFP positive area represented where the AAVs was injected. b-d Double immunofluorescence for GFP and GFAP. Note that apoE siRNAs were expressed in GFAP positive astrocytes (arrowheads). [file 13195_2020_688_MOESM2_ESM.zip › Figure S4.tif]

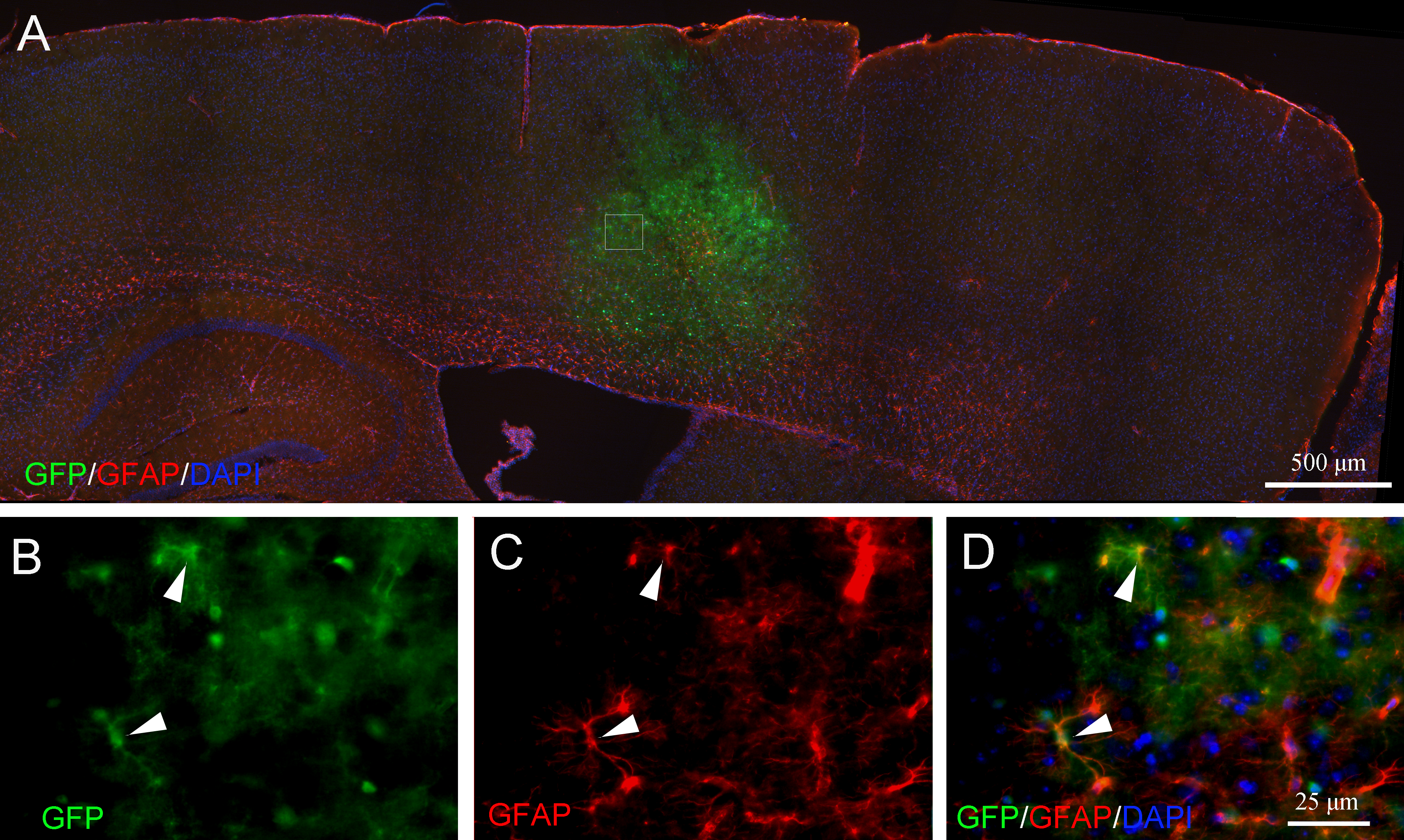

Supplement: Supplementary file 2 — Additional file 2: Fig. S1. No obvious effects of AQP4 deletion on spatial cognitive function of 3-month-old APP/PS1 mice. a, b The mean escape latency and swimming speed during the training period of the Morris water maze test. c The number of crossing the platform. d The percentage of time in the target quadrant. e The number of entries into the novel arm. f The percentage of time in the novel arm in the Y-maze. Data in S1a, b were analyzed by repeated-measures ANOVA with post hoc Student-Newman-Keuls test. Other Data were analyzed by ANOVA with post hoc Student Newman-Keuls test. Data are means ± SEM. n = 12 per group. Fig. S2. AQP4 deletion did not affect astrocyte activation in 3-month-old APP/PS1 mice. a, b Double immunofluorescence and quantification for GFAP and total-Aβ in the cortex. c, d Immunofluorescence and quantification for GS positive astrocytes in the cortex of APP/PS1 mice and AQP4−/−/APP/PS1 mice. Data are means ± SEM. n = 4 per group, two-way ANOVA with Newman-Keuls post-hoc test. Fig. S3. AQP4 polarization was impaired in the cerebral cortex of 3-month-old APP/PS1 mice. a Double immunofluorescence for AQP4 and GFAP. b Quantitative analyses of the AQP4 polarization. Data are means ± SEM. n = 4 per group, Student’s t-test. **p < 0.01; ***p < 0.001. Fig. S4. Increased astrocyte activation in the cortex in 3-month-old APP/PS1 mice and AQP4−/−/APP/PS1 mice receiving local injection of clodronate liposomes. a, b Double immunofluorescence and quantification for total-Aβ and GFAP in the cortex. Data are means ± SEM. n = 4 per group, two-way ANOVA with Newman-Keuls post-hoc test. Fig. S5. An image shows GFP expression in the cortex one month after injection of AAV encoding apoE siRNAs. a GFP positive area represented where the AAVs was injected. b-d Double immunofluorescence for GFP and GFAP. Note that apoE siRNAs were expressed in GFAP positive astrocytes (arrowheads). [file 13195_2020_688_MOESM2_ESM.zip › Figure S5.tif]
